# The use of objective structured clinical examination in dental education- a narrative review

**DOI:** 10.3389/froh.2024.1336677

**Published:** 2024-02-02

**Authors:** Mohammad Ramadan Rayyan

**Affiliations:** Prosthodontic Department, College of Medicine and Dentistry, Riyadh Elm University, Riyadh, Saudi Arabia

**Keywords:** OSCE, dental education, assessment, dental students, exam

## Abstract

The Objective Structured Clinical Examination (OSCE) is a performance-based assessment intended to assess medical students' clinical competency in a simulated, standardized environment. Because it measures the student's ability to use clinical knowledge, diagnostic skill, and decision-making, the OSCE is thought to be more objective than traditional tests. OSCE exams have been increasingly employed in dentistry schools, particularly in the last decade, and it is crucial to investigate instructors' and dental students’ experiences with this evaluation approach.

## Introduction

The Objective Structured Clinical Examination (OSCE) is a performance-based exam developed by Harden in the 1970s to assess students' competencies in relation to knowledge, skills, and attitudes in actual or simulated standardized situations (1). The OSCE is thought to be more objective than traditional exams. Rather than testing knowledge only, the OSCE evaluates the student's ability to use clinical knowledge, diagnostic skills, and decision making (2).

The evaluation of students' competency in areas such as communication, problem-solving, and decision-making can be challenging. However, OSCEs offer significant advantages in assessing these areas compared to other types of clinical exams, with higher reliability, validity, and objectivity (3).

OSCE tests are increasingly being utilized in dental schools. Moreover, many of the dental practice regulatory bodies are adopting OSCE as a method of assessment for the licensure examination (4).

The purpose of this article is to review the existing literature on the use of OSCE exams in dental schools and the benefits they provide in measuring clinical competence. A PubMed search was undertaken to find literature on the usage of OSCE exams in dental schools. “OSCE, dentistry, dental education” were the keywords used in the search and the search was restricted to English-language papers.

## The use of OSCE in dental schools

OSCE exams have been found to be effective in identifying the strengths and weaknesses of dental students and providing feedback to help them improve their clinical skills. Typically, the exams are designed to examine clinical skills and knowledge in a controlled and standardized setting. The OSCE in dentistry is designed to examine students' competencies in a variety of areas, including knowledge, skills, clinical reasoning, anamnesis, communication (both verbal and written), oral health education, and patient interaction (5).

OSCEs have been found to offer various benefits in dentistry schools, including better inter-examiner reliability, standardized evaluation, and the capacity to assess a wide range of clinical abilities. They ensure that all students are assessed on the same set of competencies and that the assessment is objective and fair. Furthermore, OSCE tests provide students with a safe and controlled setting in which to practice their clinical skills and acquire confidence in their abilities (6).

Previous research has found a link between OSCE scores and clinical and didactic performance, lending credence to the utility of OSCEs as a form of assessment (7).

OSCE tests are used in several dental schools across the world. The experience of dentistry schools in administering OSCE exams varies greatly depending on the school's resources and skills. Some schools may have specialized employees or departments to supervise the design and administration of OSCE tests, whereas others may rely on faculty or other staff members (5, 8).

A common method for administering an OSCE exam in a dentistry school is to set a series of stations, each with a particular assignment or scenario for the student to accomplish within a certain amount of time. The situations are reviewed by qualified examiners who grade students on specified criteria (9).

Standardized simulated patients are also used in some dentistry schools' OSCE tests. Standardized patients are trained actors who portray real patients with certain diseases or concerns. These patients can provide the student with a realistic and standardized experience. Simulated patients may comprise both actors and non-teaching university personnel. They are trained to play specific roles and employ scripts that have been produced for each station. To guarantee the objectivity of the test, the scripts must be extensive and comprehensive, and their confidentiality and security must constantly be ensured. Simulated patients must behave consistently with all students, including the emotions they express during the test, in order to provide a fair and objective assessment of students' skills. Actors must be prepared and trained in order to standardize their involvement, which is critical for evaluating not only the student's examination skills but also their interpersonal skills (9, 10).

The administration of OSCE adheres to fundamental principles and allows flexibility in its implementation based on targeted learning outcomes and tested competencies. The design of OSCE levels, the selection of standard cases or situations, and the assessment criteria can be tailored to emphasize specialized clinical skills or competencies related to one or more learning domains (10). The review of the literature showed that OSCE exams are regarded as an effective tool for assessing clinical competence in different dental specialties, such as restorative dentistry, prosthodontics, endodontics, periodontics, oral surgery, oral medicine, oral radiology, orthodontics, and pediatric dentistry (10–15).

For instance, in one published OSCE scenario in periodontology, the 21-year-old actress played the role of a complex patient presenting with necrotizing gingivitis (NG). The patient was given a script to follow, and prior training to clarify her doubts and establish a pattern that was realistic. The teachers assessed the students' reaction and management of this situation. Students were graded on communication skills, diagnostic judgment, treatment strategies, preventive measures, and ethical considerations. They were carefully evaluated for their performance on a 47-point scale checklist, with students needing at least 50% of the total marks to pass this challenging level with a comprehensive test of their ability to manage the patient and the case (10).

Another example of the use of OSCE in dental schools is reported by Fields et al. (14) who introduced OSCE to an advanced orthodontic education program to evaluate its impact on the curriculum. To formulate the examination's content, 60 orthodontic practitioners were contacted to assess which clinical skills are essential for an entry-level practitioner to possess. Thirteen of the eighteen crucial clinical skills were assessed by the OSCE in the domains of orthodontic technique, clinical evaluation and synthesis, and diagnosis (14).

Höfer et al., (15) introduced OSCE as a method of assessment in a problem-based learning (PBL) curriculum. Students of Cranio-Maxillo-Facial Surgery practical course were presented with clinical scenarios at 10 stations as they rotated through them. These stations included trauma (managing a fractured mandible or malar bone, managing intraoral or extraoral bleeding), practical knowledge (ligature of 8, probe biopsy, intravenous line, ligature with wire, realigning a mandible that had become dislocated), diagnosis (describing intraoral tumors, x-ray, CT-scan, craniofacial examination), and oncology (describing intraoral tumours, for example, excision). An examiner used a standardized multi-item checklist to observe and evaluate the students' performance during each five-minute station (15).

In dental schools, OSCE can be utilized to assess competence in a specific subject as well as multiple domains and specialties. Assessing several domains at each station was reported to be more realistic and helped students understand the relevance of OSCE (13). An example is reported by Manogue and Brown. They prepared a total of 17 stations in the areas of conservative dentistry, periodontology and prosthodontics. Across the subjects, 9 stations tested knowledge, 4 tested procedures, 5 tested clinical reasoning, 3 tested history taking, 4 tested communication and 4 tested techniques. One station also tested the provision of oral health education. 15 stations had 5-minute tasks and the remaining 2 had 10-minute tasks. 4 stations involved the incorporation of simulated patients, e.g., where a medical history was to be taken (13).

Checklists were previously used to assess student performance in tasks such as problem-solving, but this approach has drawbacks because it simply allows examiners to watch rather than analyze behaviors. Rating scales that use a Likert-type scale to consider performance across multiple areas have been advocated as a more valid technique for analyzing candidate behaviors. However, training examiners to use these scales can take time, thus e-learning aids such as videos and training packages may be a more practical option (16).

In addition to the actual administration of the OSCE exam, dental schools may also invest significant resources in the development of the exam. This may include the creation of standardized scenarios or cases, the development of assessment rubrics, and the training of examiners (17).

The findings of OSCE can offer educationists with useful information when establishing clinical dentistry education programs. Some dental schools now include comments from OSCE exams into their curriculum. Students may receive a thorough report on their performance, including strengths and shortcomings, following the exam to assist them in identifying areas for improvement. Students and professors found a combination of written and auditory feedback approaches to be more beneficial (18). The feedback offered should characterize test performance based on the core domains of learning that the test attempts to examine, as well as include the resources needed to implement individualized improvements at an individual level. This form of feedback is very beneficial to students because it allows them to focus their learning efforts on areas that require development (19).

## Student experiences with OSCE

Many dentistry schools have students evaluate their OSCE tests. This feedback is utilized to improve the exam's design, delivery, and evaluation. Students frequently seek more time, better instructions, and more opportunity for feedback and guidance from faculty (9).

Student opinions and experiences with OSCE exams in dental schools vary. Overall, student feedback has been positive, with many students recognizing the value of the OSCE in assessing clinical skills and appreciating the practical approach of OSCE exams, which tests their ability to apply theoretical knowledge in a clinical setting (19). The structured format of the OSCE exams also ensures that all students are tested on the same skills and given equal opportunities to showcase their skills and knowledge. Several studies have reported that majority of dental undergraduate respondents stated they would choose to have a similar format of OSCE assessment in future which reflects student's support for this type of assessment (8).

Students from different dental schools reported that they felt OSCE went beyond fact memorization, required knowledge application, demanded critical thinking and problem-solving abilities, and was an authentic educational experience (20).

Some students, however, found the OSCE exams scary, unpleasant, and anxiety-inducing, especially when there are multiple stations with limited time to complete each task. The timed, interactive components of the OSCE as well as the examiners' continuous observation and monitoring may be the cause of the higher anxiety levels that have been reported. According to one the study, there is no reason to stop using OSCEs as an assessment tool for dental undergraduates, because the reported levels of anxiety among students did not predict their OSCE results (21).

Furthermore, some students thought that the scenarios did not always accurately reflect the clinical situations they encountered in practice. This seems to be largely affected by the type of OSCE stations. Students reported that the use of Phanom heads and acrylic models to simulate the chairside clinical situation have many drawbacks including the lack of clinical authenticity, and lack of communication skills testing. Simulated patients help to overcome these drawbacks. Nevertheless, simulated patients scenarios are usually limited to non-invasive procedures which does not test manual dexterity and is not effective in assessing clinical operative skills (22).

Some students also believe that the OSCE exams do not reflect their true ability because they are timed and do not always allow for the creativity and flexibility required in real-life clinical settings (23). Combining OSCE with other assessment methods was recommended to overcome these drawbacks and improve assessment validity and student's learning experience (16, 24).

## OSCE's limitations

Despite the numerous advantages of OSCE exams, there are some drawbacks that must be addressed. One of the most significant constraints is the expense and time required to put up an OSCE exam. Another limitation is the possibility of students memorizing the situations and becoming disinterested in the exam. Furthermore, OSCE exams may not adequately depict the complicated nature of clinical practice or assess students' competence to engage with real patients (25).

Simulated patient OSCE stations can be challenging due to several logistical issues. These are stations that take a great deal of planning and preparation: choosing and training the actor, setting up a large area with multiple rooms, calibrating the rating system, hiring support staff to operate the computer, and creating any additional tests that might be required (x-rays, periodontal charts, study models, etc.) (13).

Some educators have expressed worry that the fragmentation of complex clinical situations into short OSCE stations could lead to a loss of validity. In order to achieve thorough and efficient assessment, educators investigated the benefit of combining OSCE with other assessment techniques. Some authors suggested that combining the Objective Structured Clinical Examination (OSCE) with other assessment methods such as Direct Observation of Procedural Skills (DOPs) and Mini-Clinical Evaluation Exercise (mini-Cex) can be an effective and feasible evaluation tool for clinical dentistry. DOPs assesses the students' operational abilities, while mini-Cex evaluates their diagnosis and treatment abilities in real-world clinical settings (24, 26).

Some quantitative studies show that examiners frequently differ in their OSCE scores. This could pose a significant risk to the reliability of assessment results and compromise the validity of the entire testing procedure (27, 28). To reduce this risk and ensure the validity of exam results, candidates should complete a sufficient number of stations, particularly with multiple examiners. Examiner differences could be lessened by rigorous preparations, training and displaying various scoring patterns (29).

Students frequently complain about a lack of preparation for OSCE tests. While students' OSCE preparation resources are contradictory, alternative activities such as pro-OSCEs with peer feedback and the use of online resources, including serious games, are growing. Serious games are non-recreational games that have been shown to be at least as successful as more traditional resources for enhancing knowledge, skills, and overall satisfaction. Because of its immersive experience and round-the-clock availability, serious gaming may be useful for OSCE preparation (30).

## Virtual OSCE

The Remote Objective Structured Clinical Examination, or Virtual OSCE, uses telecommunication technologies to perform clinical assessments remotely. During the COVID-19 pandemic, the use of virtual OSCE has become increasingly important for evaluating dental students’ clinical competencies while adhering to social distancing guidelines (31). The exam is conducted online via video conferencing software, with students interacting with standardized patients in another location. The standardized patients could be actors or genuine patients who have been trained to imitate clinical events, giving students the opportunity to demonstrate their knowledge, skills, and patient contact abilities in a realistic context. The exam is conducted using defined methods and assessment tools to assure the validity and reliability of the remote OSCE. The utilization of digital tools like high-resolution cameras and real-time video conferencing software also contributes to a more realistic and immersive experience for both students and standardized patients (32–34).

Remote OSCEs can be very useful for assessing students' communication skills with patients, which is an important feature of dental practice. Furthermore, they can help to reduce the logistical challenges and costs associated with traditional in-person clinical exams, such as the need for physical space and standardized patients (35, 36).

Virtual OSCE was well-received by both students and examiners and found it to be as engaging and as interactive as in-person teaching. Current evidence showed moderate agreement of virtual OSCE with on-site clinical assessments. However, this was based on studies with low methodological quality small sample sizes. Thus, additional research is necessary to further investigate the efficacy and validity of virtual OSCE in medical teaching including dentistry (37).

## Peer OSCE

A Peer OSCE is a technique used to evaluate medical students and healthcare workers by their peers. It entails watching and giving comments to colleagues as they conduct clinical activities on standardized patients. This method has a number of advantages, including the ability to receive constructive comments from others with similar knowledge and skill levels. This feedback can help trainees improve their clinical abilities and think critically about how they provide care to patients. Furthermore, the collaborative character of peer OSCEs can build a supportive learning atmosphere, promote teamwork, and boost learners' confidence (38).

Peer OSCE is also inexpensive as it needs few resources and is simple to include into existing courses. Overall, the benefits of peer OSCEs include feedback, improved learning, teamwork, increased confidence, and cost-effectiveness. Because of these advantages, peer OSCEs have the potential to be a beneficial tool for measuring and enhancing clinical skills among dental students (39).

One study compared the perceived value of dental students' feedback on their performance in OSCE from either a faculty member or a peer student. Students perceived value in the feedback from both faculty and peers and believed it enhanced their skills. However, students rated faculty feedback significantly higher. Peer feedback on nontechnical clinical competency assessments could be a valuable tool for the learning environment, though not as a substitute for faculty feedback (39).

To obtain the desired benefits of peer feedback, the students need a lot of training on the administration of OSCE with emphasis on how to give constructive feedback in a positive environment. Feedback given in a negative way may influence the student's performance in subsequent stations (40).

## Conclusion

In conclusion, various research and reports have demonstrated that OSCEs are an effective technique for evaluating clinical competency in dental education. They offer a dependable and valid way for assessing students' clinical skills and identifying areas for improvement. The use of OSCE exams in dentistry schools ensures that students are evaluated in a standardized and objective manner, as well as that students can practice their clinical skills in a safe and controlled environment. Although there are certain difficulties to using OSCE tests, the benefits greatly exceed the disadvantages. To ensure the quality of dental education and foster the development of competent dental practitioners, dental schools should continue to incorporate OSCE assessments within their curriculum. Dental schools should also use student input to improve the design and delivery of their OSCE exams, ensuring that they are a useful tool for assessing students' clinical knowledge and skills.
